# The novel *CFTR* haplotype E583G/F508del in CFTR-related disorder

**DOI:** 10.1007/s11033-024-09732-x

**Published:** 2024-07-25

**Authors:** Elisa De Paolis, Bruno Tilocca, Riccardo Inchingolo, Carla Lombardi, Alessia Perrucci, Giulia Maneri, Paola Roncada, Francesco Varone, Richeldi Luca, Andrea Urbani, Angelo Minucci, Concetta Santonocito

**Affiliations:** 1 Departmental Unit of Molecular and Genomic Diagnostics, Genomics Core Facility, Gemelli Science and Technology Park (G-STeP), Fondazione Policlinico Universitario A. Gemelli IRCCS, Rome, 00168 Italy; 2 Clinical Chemistry, Biochemistry and Molecular Biology Operations (UOC), Fondazione Policlinico Universitario A. Gemelli IRCCS, Rome, Italy; 3 Department of Health Science, University “Magna Graecia” of Catanzaro, Catanzaro, 88100 Italy; 4 Pulmonary Medicine Unit, Department of Medical and Surgical Sciences, Fondazione Policlinico Universitario A. Gemelli IRCCS, Rome, Italy; 5 Catholic University of Sacread Heart, Rome, 1-00168 Italy; 6 Department of Basic Biotechnological Sciences, Intensivological and Perioperative Clinics, Catholic University of Sacred Heart, Largo Agostino Gemelli,, Rome, 00168 Italy

**Keywords:** *CFTR*, CFTR-RD, Variants of unknown significance, Next generation sequencing

## Abstract

**Background:**

CFTR-related disorder (CFTR-RD) is a clinical entity associated to complex diagnostic paths and newly upgraded standard of care. In CFTR-RD, *CFTR* genotyping represents a diagnostic surrogate marker. In case of novel haplotype, the diagnosis could represents an area of concern. We described the molecular evaluation of the rare *CFTR* variant E583G identified *in trans* with the F508del in a novel haplotype.

**Methods and results:**

An adult woman was referred to our pulmonary unit for persistent respiratory symptoms. *CFTR* Next Generation Sequencing was performed to evaluate full-gene mutational status. The variant identified was evaluated for its pathogenicity integrating clinical evidences with dedicated bioinformatics analyses. Clinical evaluation of patient matched with a mono-organ CFTR-RD diagnosis. Genotyping revealed the novel *CFTR* haplotype F508del/E583G. Multiple evidences of a deleterious effect of the *CFTR* E583G rare variant emerged from the bioinformatics analyses performed.

**Conclusions:**

Guidelines for CFTR-RD are available with the purpose of harmonizing clinical and molecular investigations. In such context, the identification of novel *CFTR* haplotype need to a deeper evaluation with a combination of skills. The novel E583G variant could be considered of clinical interest and overall a CFTR-RD Variants of Varying Clinical Consequences.

## Background

Cystic fibrosis (CF, OMIM 219700) is one of the most common autosomal recessive diseases among Caucasians [1]. It is known to be a childhood multisystem disorder caused by pathogenic variants in the Cystic Fibrosis Transmembrane Conductance Regulator (*CFTR*) gene, encoding for a chloride and bicarbonate channel expressed in the apical membrane of epithelial cells [2, 3]. Different *CFTR* variants lead to heterogeneous degrees of protein dysfunction with multiple clinical phenotypes. Approximately 60% of patients are identified within the first year of life and up to 90% by age 8 years [4]. A small percentage of adults presents a disease phenotype known as CFTR-related disorder (CFTR-RD) defined as “a clinical entity associated with CFTR dysfunction that does not fulfil the diagnostic criteria for CF” [5]. Individuals with CFTR-RD show a late onset with variable symptoms generally involving a single organ, typically the respiratory system, with a negative or inconclusive sweat test [6, 7]. While the diagnosis of CF is straightforward, the prompt identification of CFTR-RD could still represent a clinical challenge. Formerly, a diagnosis of CFTR-RD may be posed in a symptomatic patient carrier of two *CFTR* variants shown to reduce CFTR function, with one known to be CF-causing variant [8]. A growing number of CFTR-RD clinical and experimental data become available in the last decades mainly due to the wider use of full *CFTR* gene sequencing. This allowed the detection of rare *CFTR* variants revealing a large number of Variants of Unknown Significance (VUS) with difficult interpretation, especially in the context of CF-related symptomatology [9].

We report the assessment of pathogenicity of the p.(Glu583Gly) *CFTR* variant detected in compound heterozygosis with the common p.(Phe508del) mutation. The novel haplotype has been identified in a symptomatic patient with clinical manifestations that trace CFTR-RD. We described the clinical profile of the patient and elucidated the impact of the newly identified variant.

## Case presentation

A never-smoking 56-year-old white female was admitted to our hospital in 2019 with persistent respiratory symptoms and purulent cough, low-grade fever, and dyspnea. She presented a personal history of bronchial asthma and chronic sinusitis since childhood with a negative sweat test. Lung function tests showed severe obstructive deficit (FVC: 55%; FEV1: 33%; FEV1/FVC 51.04%). Diagnosis of bilateral bronchiectasis, poorly controlled obstructive lung disease, and Allergic Broncho Pulmonary Aspergillosis were made. The family history was negative for CF. The patient began respiratory rehabilitation treatment with T-Pep twice a day aimed at promoting the elimination of tracheobronchial secretions and inhalation therapy with two bronchodilators. Her clinical manifestations were inconclusive for a definitive diagnosis of CF. She was addressed to CFTR genetic test and successively, the molecular investigation was also extended to her asymptomatic son of 19 years old for familial screening purpose. DNA was extracted from whole blood samples using the QIAmp DNA Mini kit on Qiacube instrument (Qiagen). The Devyser CFTR Next Generation Sequencing (NGS) assay (DEVYSER) coupled with the CE-IVD Amplicon Suite Software v2.0 (SmartSeq) were used for the *CFTR* full gene sequencing. Variants were reported according to the Human Genome Variation Sequence systematic nomenclature (NM_000492.4). The variants final classification were obtained according to the American College of Medical Genetics and Genomics guidelines. Data integration of several population and mutational databases (last access May 2024) was performed: dbSNP (https://www.ncbi.nlm.nih.gov/snp/), 1000 Genomes (http://www.internationalgenome.org/), GnomAD (https://gnomad.broadinstitute.org/), ClinVar (http://www.ncbi.nlm.nih.gov/clinvar/), LOVD (https://www.lovd.nl/), Human Gene Mutation Database (http://www.hgmd.cf.ac.uk/ac/index.php), CFTR2 (https://cftr2.org/), CFTR-France (https://cftr.iurc.montp.inserm.fr/cftr), CFTR mutation database (http://www.genet.sickkids.on.ca/Home.html). The identified genetic variant has been evaluated through the implementation of multiple bioinformatic tools, namely MobyDetails, dbNSFP, and BayesDel. MobiDetails is an annotation platform for DNA variations; it accounts for both exonic and intronic sequences, retrieving annotation information from multiple sources including population frequencies, splicing predictors, and inferred functionalities [10]. dbNSFP is a database suited for functional prediction and annotation of non-synonymous single-nucleotide variants in the human genome. This relies on two independent prediction and scoring systems based, in turn, on 43 and 9 diverse algorithms, respectively besides recalling functional information by large cohort studies projects [11]. BayesDel is a pathogenicity scoring platform working with SNVs and small insertions and/or deletions onto coding and non-coding sequences. The platform outputs a score relative to the pathogenic potential of the queried variants [12]. The impact of the novel variant on protein structure and/or function was assessed *in silico* complementing a plurality of bioinformatics META tools such as VARSOME and REVEL (last access, May 2024). VARSOME is a hub collecting data from multiple databases enabling browsing and sharing of novel variants [13]. REVEL platform predicts the pathogenicity of missense variants through a scoring system which implements multiple independent tools, enabling the discovery of pathogenic and rare neutral missense variants [14]. Altogether, above tools integrate multiple independent computational algorithms. Moreover, protein variants have been subjected to protein-protein interaction (PPI) analysis through dedicated bioinformatic tools. PSOPIA predicts protein-protein interactions exploiting: (i) sequence similarities to a known interacting protein pair; (ii) statistical propensities of domain pairs observed in interacting proteins, and (iii) a sum of edge weights along the shortest path between homologous proteins in a PPI network [15]. Complimentarily, Tri-Tool predict PPI based on the HIPPIE (Human Integrated Protein-Protein Interaction rEference) dataset. Besides, its computing integrates information on the pseudo-Amino Acid Composition (PseAAC), hydrophobicity, hydrophilicity, side-chain mass, and the Electron-ion Interaction Potential (EIIP), a descriptor of long-range interaction properties that contribute considerably to protein binding specificity [16].

## Discussion and conclusions

**Fig. 1:**
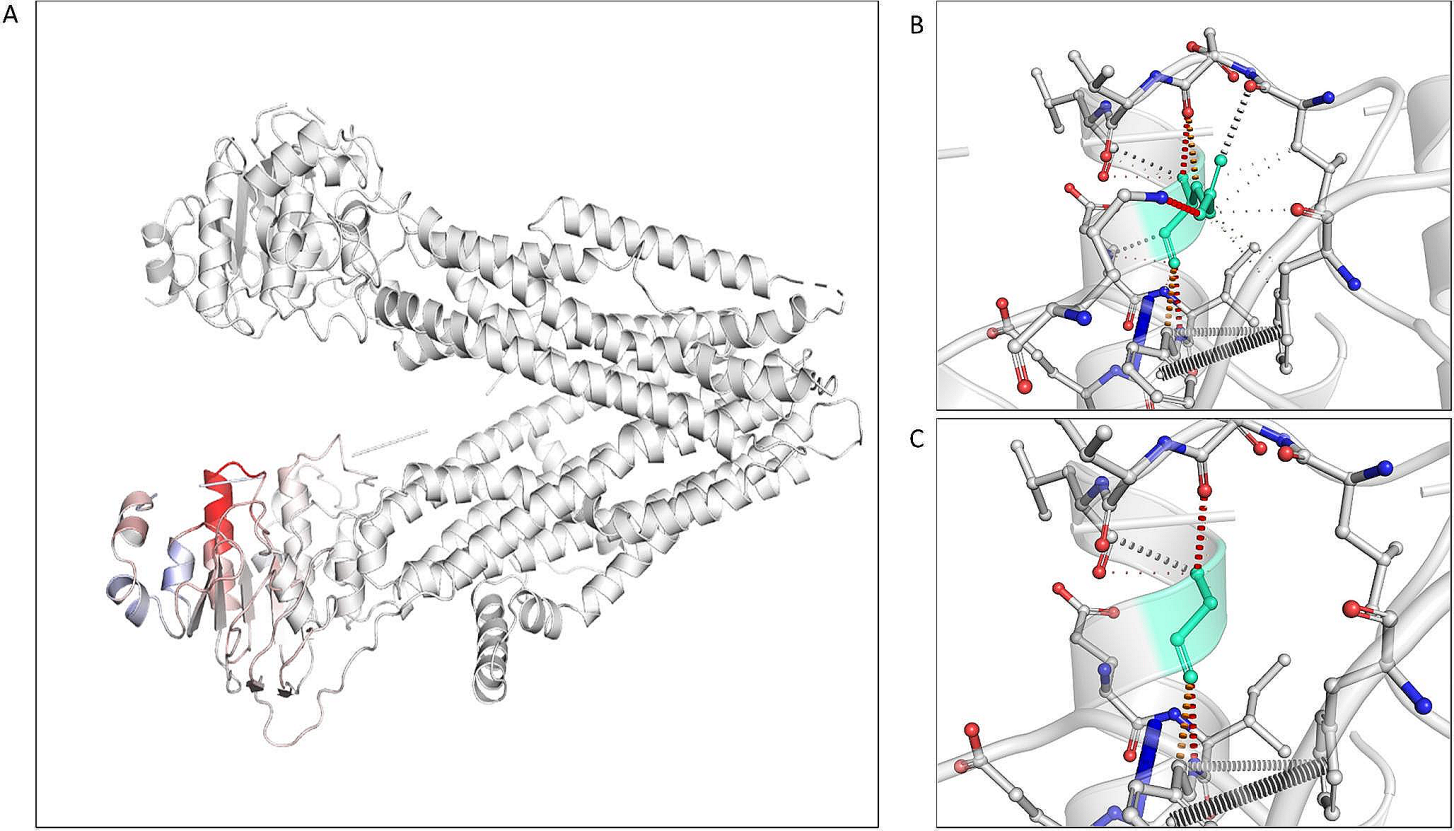
Tridimensional structure of the CFTR protein as of the 5AUK structure in PDB data repository. **(A**) Protein moieties are coloured according to the vibrational entropy change upon mutation. Blue shades are representative of a rigidification of the structure while red shades indicate a gain in flexibility. (**B**) Interatomic interaction of the wild type and (**C**) mutant protein. *Wild-type* and mutant residues are coloured in light green and are represented along with the surrounding residues which are involved in any type of interaction

The *CFTR* gene sequencing analysis revealed the presence of two gene variants. The pathogenic variant *c.1521_1523delCTT*, p.(Phe508del) was detected with a Variant Allele Frequency (VAF%) of 48%, together with the novel *c.1748 A > G*, p.(Glu583Gly) missense variant with a VAF% of 45%. Poly-TG/poly-T evaluation resulted in TG10-T9/TG11-T7 repeats. The *CFTR* alteration p.(Glu583Gly) is located in the exon 13 and it is annotated in dbSNPs database (rs1421257199) and GnomAD as a rare alteration (1/152110 alleles, 6.57^e-6^ of frequency). It is not present in the main *CFTR* mutational databases as CFTR2 and CFTR-France. This alteration was detected only in the patient here described among the large cohort of subjects who underwent *CFTR* testing in our Institution. Given the lack of clinical annotation, the variant was considered a VUS at the time of the present paper. The *CFTR* sequencing analysis of the proband’s son detected only the novel *CFTR* variant, proving the *in trans* status of the alleles. To the best of our knowledge, the identified haplotype was not previously reported. In order to estimate the effect of the variant, we set out multiple *in silico* analyses. Table 1 summarizes the scores from individual predictors and meta-predictors. All the bioinformatics META tools calculated a pathogenic effect of the p.(Glu583Gly) substitution while from the 17 individual tools, 12 showed a prediction of pathogenicity. A deeper investigation of the *wild-type* and mutated protein underlines that the residue Glu583 is conserved at 98% among the CFTR orthologs, with the sole CFTR protein sequence replacing it with a Gly-residue in Caenorhabditis elegans. Onto the CFTR structure, the substitution is predicted to be included in an α-helix motif of NBD1, with a score of 0.86. The *wild-type* domain conservativeness is estimated in 11.53% of the homologs, whereas, the mutant domain is computed in 4.47% of the homologous proteins. Effects of the p.(Glu583Gly) substitution in the tertiary structure have been assessed on the high definition 3D-structure available in the Protein DataBank (5AUK). The 3D model confirmed previous prediction collocating both Glu583 and Gly583 onto the α-helice of the NBD1. Specifically, the residue falls in the turn N4 of the α-helice, comprising a total of 15 residues. Mutant residue has less affinity for the middle of helices. Prediction of the thermodynamic stability of the protein upon substitution reveal a weak destabilizing effect for p.(Glu583Gly) variant with a ΔΔG: -0.899 kcal/mol. Substitution of glutamic acid-residue with glycine is likely to increase the flexibility of the NBD1 region. The increased structural flexibility is also confirmed by the ΔVibrational Entropy Energy computed between *wild-type* and mutant variant as + 0.951 kcal.mol-1 K-1. A visual representation of substitution effects on the CFTR tertiary structure, computed as means of the ΔVibrational Entropy Energy, is reported in Fig. 1, panel A. Both *wild-type* and the mutant Gly583 are predicted to be buried with respect to the solvent accessibility. The two residues involved in the aminoacid change have a different polarity, which could interfere with hydrogen-binding capabilities of the whole NBD1 region. Indeed, the different electronic features of glutamic acid and glycine result in diversified ionic bonds and/or inter- or intra-molecular interactions the two protein variants (*i.e. wild-type* and mutant) are capable of, as shown in Fig. 1, panels B-C.

**Table 1:** Bioinformatics prediction of pathogenicity. List of meta and individual *in silico* tools applied for the prediction of pathogenicity of the *CFTR* p.(Glu583Gly) variant. For each tool, output score and prediction were reported. *Larger score corresponds to more likely damaging effect. **Smaller score corresponds to more likely damaging effect

Type of score	Tool	Values	Prediction (threshold for damaging)
Meta	REVEL	0.775	Damaging [> 0.5]
Meta	Meta SVM	0.2705	Damaging [> 0]
Meta	Meta LR	0.7171	Damaging [> 0.5]
Meta	BayesDel	0.384	Damaging [≥ 0.09]
Meta	VARSOME	-	Pathogenic moderate
Individual	SIFT	0.691	Tolerated [< 0.05]
Individual	MutPred2	0.759	Probably Damaging [0–1]*
Individual	MVP	0.98	Damaging [≥ 0.75]
Individual	Polyphen 2 HumDiv	0.981	Probably Damaging [≥ 0.957]
Individual	Polyphen 2 HumVar	0.951	Probably Damaging [≥ 0.909]
Individual	Fathmm	-3.12	Damaging [≤ -1.5]
Individual	Mutation Assessior	-0.215	Neutral [5.17–6.49]*
Individual	PROVEAN	1.61	Neutral [-14–14]**
Individual	LRT	0	Damaging [0–1]**
Individual	M-CAP	0.554	Damaging [0–1]*
Individual	DEOGEN2	0.230	Tolerated [> 0.5]
Individual	LIST-S2	0.936	Damaging [> 0.85]
Individual	MutationTaster	-	Disease causing
Individual	PrimateAI	0.627	Tolerated [> 0.80]
Individual	AlphaMissense	0.599	Likely Pathogenic [> 0.564]
Individual	ClinPred	0.973	Damaging [≥ 0.5]
Individual	Mistic	0.71	Damaging [≥ 0.5]

Diagnosis of CF can be established if biomarkers of CFTR protein activity show dysfunction, in association with compatible *CFTR* genotype. Clinical conditions that not fully meet diagnostic criteria of CF could be defined as RD-CFTR. At present, the identification of CFTR-RD patients are critical points with certain degree of misdiagnosis. In this context, the detection of *CFTR* VUS or Variants of Varying Clinical Consequences (VVCC) with residual CFTR function worsens the clinical framework due to the lack of clear molecular data [17]. Overall, criteria for RD-CFTR include: (i) CF symptoms in one or multiple organ systems; (ii) normal or borderline CFTR function tests, mainly sweat chloride, and (iii) two *CFTR* variants shown protein gene function reduction, with one of these known to be a CF-causing mutation [2, 5, 8, 18]. However, the line between the definitions of a CF- and CFTR-RD-causing mutation is not absolute. In CFTR-RD is observed one severe *CFTR* variant (class I-III) and one uncommon variant (or an abnormal number of trinucleotide repeats) [1, 19]. We characterized the *CFTR* p.(Glu583Gly) variant identified in the context of a CFTR-RD phenotype in compound heterozygous with the class II pathogenic p.(Phe508del) mutation. The variant could be considered a deleterious alteration according to the evidence of multiple algorithms employed. In this perspective, obtaining supporting data from a plurality of recognized bioinformatics tools enhances the accuracy of the presented outcomes, enabling the fair inference of the structural and functional properties of this rare variant [20–23]. Minor discrepancies in the scoring values are, to some extent, expected to acknowledge the different computational approaches. On the other hand, the overall computing performed shares the conclusion of a damaging variant due to the structural rearrangement triggered by the amino acid substitution. The literature review and the main *CFTR* mutational databases revealed no prior report of the variant. The glutamic acid to glycine substitution is a non-conservative change and the replacement involves an acidic and negatively charged amino acid with a small, nonpolar, and neutral residue. This alteration may affect the charge distribution, local structure, and stability of the domain. Accordingly, using an in vitro NBD1 structural complementation assays to assess folding and stability, Mendoza et al. described as the p.(Glu583Gly) variant, coupled with the p.(Phe508del), decreased the yield of soluble NBD1, worsening its folding in both the isolated domain and the full-length *CFTR* [24]. Additionally, the Glu583 residue localize in the NBD1 region of the CFTR protein which is critical for the ATP binding and the channel activation (probably class III).

## Conclusions

According to international standards, clinical, epidemiological, and functional data are necessary for considering a *CFTR* variant as CF causing [8, 18]. Overall, the CFTR p.(Glu583Gly) variant is likely to be of clinical significance *in trans* status with the pathogenic variant p.(Phe508del). The novel *CFTR* haplotype is suggestive to be CFTR-RD causing also taking into account the clinical manifestations. The clinical evidences of multiple illnesses linked to CF together with the *in silico* analysis indicate for the variant p.(Glu583Gly) a classification as a VVCC CFTR-RD-causing in the genotype here described (CF/VVCC). While further information derived from experimental studies or from the description of the same variant in homozygosis or the same haplotype in other affected subjects are needed to consider the novel variant as CF-causing.

Individuals with CFTR-RD have a longer life expectancy, but the long-term effects of the disease remain unknown. Efficient diagnosis and adopted therapy certainly improve the quality of life of patients and their families.

## Data Availability

No datasets were generated or analysed during the current study.
